# Investigation of the Prevalence of *Fasciola hepatica* in Small Ruminants in the Siirt Region, Turkey

**Published:** 2018

**Authors:** Özgür Yaşar ÇELİK, Burçak ASLAN ÇELİK

**Affiliations:** 1. Dept. of Internal Medicine, Faculty of Veterinary Medicine, Siirt University, Siirt, Turkey; 2. Dept. of Parasitology, Faculty of Veterinary Medicine, Siirt University, Siirt, Turkey

**Keywords:** Sheep, Hair Goat, *Fasciola hepatica*, Turkey

## Abstract

**Background:**

Fasciolosis is a disease of the liver caused by trematodes in the family of Fasciolidae, particularly by *Fasciola hepatica* and *Fasciola gigantica*. The aim of this study was to investigate the prevalence of *F. hepatica* in sheep using the ELISA method, and in hair goats by post-mortem liver examination in the Siirt region, Turkey.

**Methods:**

This study was conducted between Feb–Sep 2018. Five ml of blood samples were taken from the jugular veins of 320 sheep, which were selected from various locations of Siirt region by random sampling method. Fasciolosis seroprevalence in sheep was investigated by the ELISA method, using commercial kits (BIOK 211-Monoscreen AB ELISA *F. hepatica* test). In order to determine the prevalence of fasciolosis in hair goats, 580 slaughtered goats were examined for *F. hepatica* by incisions in the liver, gallbladder, and bile ducts.

**Results:**

While 24 (7.50%) sheep were seropositive, 296 (92.50%) were seronegative. Regarding the hair goats, on the other hand, 82 (14.14%) were positive, while 498 (85.86%) were negative.

**Conclusion:**

*F. hepatica* infection causes significant economic losses due to the destruction of the liver in small ruminants. Considering zoonotic properties of the disease, it has been concluded that the necessary measures should be taken and anti-helminthic drugs should be applied to the animals that come out of the pasture. Furthermore, periodic examinations should be conducted, and the breeders should be informed about the disease to raise awareness.

## Introduction

In various herbivores such as sheep, goats, and cattle, the species of fasciola have zoonotic characters as trematodes that are located in the liver bile ducts (1, 2). Fasciolosis is primarily a liver disease caused by trematodes in the family of Fasciolidae, particularly by *Fasciola hepatica* and *F. gigantica*, and various snails of Lymnaeidae family act as intermediate hosts of these parasites that constitute the disease (3, 4).

Parasitic infections adversely affect the animals by influencing the food intake and digestion (5–7). The disease leads to significant economic losses by causing high mortality rates and morbidity in sheep and goats in endemic areas and leads to increased sensitivity to secondary infections, and increased costs that arise due to prevention methods (2).

Post-mortem inspection of animals with parasitic infestations reveal that large sections of the liver are affected. Such a situation may result in the partial or complete destruction of the liver and cause significant economic losses (8, 9).

Clinical and postmortem findings, stool examination methods, biochemical analyses, imaging techniques, and serological methods are used in the diagnosis of fasciolosis (10). A fecal examination is amongst the most common methods used to diagnose the disease, but the eggs can only be seen in the stool at the mature period of the parasite. The earliest diagnosis of the parasite with fecal examination is only possible at 13^th^–14^th^ weeks (4). As a result, alternative serological methods for early diagnosis of the parasite were developed (4, 10).

The aim of this study was to investigate the prevalence of *F. hepatica* in sheep using the ELISA method, and in hair goats by post-mortem liver examination in the Siirt region, Turkey.

## Materials and Methods

### Study area

**Fig. 1:**
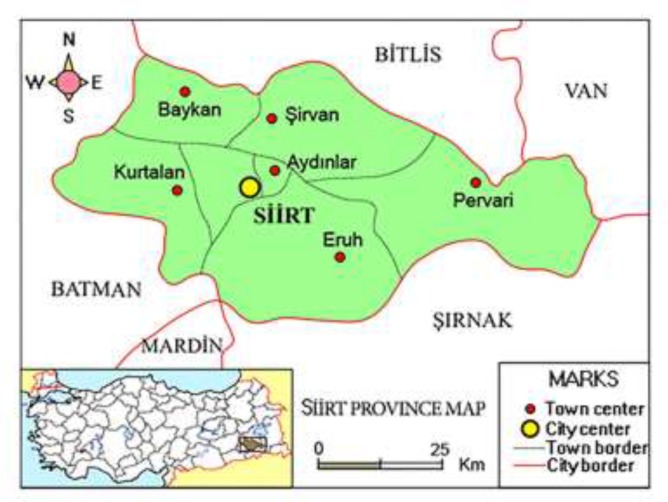
Siirt province map

The Siirt Province (Fig. 1) lies in the sub-humid climate region according to the Thorntwaite Climate Classification (C2, B’3,s2,b’2). The annual precipitation in the Province is 715.4 mm. The average highest and lowest temperatures are between 36.9 °C and 18.9 °C in summer, and 8.7 °C and −0.5 °C in winter. There are frequent water shortages during the summer (11).

### Sample collection

This study was conducted between Feb–Sep 2018. Five ml of blood samples were taken from the jugular veins of 320 sheep, which were selected from various locations of Siirt region by random sampling method. The samples were centrifuged for 10 min at 3000 rpm in order to obtain the serums. The obtained serums were stored at −20 °C until further analyzes were performed. Fasciolosis seroprevalence in sheep was investigated by the ELISA method, using commercial kits (BIOK 211-Monoscreen AB ELISA *F. hepatica* test).

In order to determine the prevalence of fasciolosis in hair goats, 580 slaughtered goats were examined for *F. hepatica* by incisions in the liver, gallbladder, and bile ducts.

### Ethical approval

Ethical approval for this study was obtained from the Siirt University Local Ethics Committee for Animal Experiments (DEHAM). (Approval Date and Numer: 10.02.2017-2017/07)

## Results

While 24 (7.50%) sheep were seropositive, 296 (92.50%) were seronegative. Regarding the goats, on the other hand, 82 (14.14%) were positive, while 498 (85.86%) were negative (Table 1).

**Table 1:** prevalence of *Fasciola hepatica* in small ruminants in Siirt Province, Turkey

***Species***	***Examined No.***	***Positive***		***Negative***	
		***n***	***%***	***n***	***%***
Sheep	320	24	7.50	296	92.50
Goat	580	82	14.14	498	85.86

## Discussion

Liver trematodes of domestic ruminants constitute an important parasite group in the world because of yield losses and significant economic losses due to the destruction of the infected liver (12).

Studies conducted in various countries have revealed that the prevalence of fasciolosis varies from region to region, and is influenced by environmental conditions, animal species and breeding method, but it is reported that the disease range for sheep is between 0.7%–29.4% and 0.13%–2.02% for goats (13).

Turkey has suitable geographical features in terms of both climatic and ecological factors for the prevalence of the Fasciola types. However, studies regarding the prevalence of the fasciolosis in a limited number (14).

In the studies on the distribution of small ruminant fasciolosis in different regions of Turkey; Antalya, Van, Trakya, Kars, Sivas, Tatvan, Elazığ, Malatya, Adana, Samsun, Sinop, Tokat and Hakkari were found to have the ratios of 29.1%, 15.60%, 3.99%, 9.4%, 5.97%, 72.6%, 1.6%, 4.42%, 6.6%, 32.4%, 25.4%, 34.9%, and 41.21%, respectively (2, 8, 12, 13, 15–21).

As a result of this study, the prevalence of disease in sheep and goats was found 7.50% and 14.14%, respectively. When compared with other studies in Turkey, the results obtained in our study were lower than the studies of some researches (12, 15–17, 20, 21), while it was higher than the studies of others (2, 8, 13, 18, 19).

When this study and the literature results are compared, the emerging variation may be explained by the climatic differences, the annual rains, the animal raising methods (intensive, semi-intensive, extensive), and pasture grazing time.

## Conclusion

*F. hepatica* infection causes significant economic losses due to the destruction of the liver in small ruminants. Considering zoonotic properties of the disease, it has been concluded that the necessary measures should be taken, and anti-helminthic drugs should be applied to the animals that come out of the pasture. Furthermore, periodic examinations should be conducted, and the breeders should be informed about the disease to raise awareness.

## References

[1] Ahmed E Markvichitr K Tumwasorn S Koonawootrittriron S Choothesa A Jittapalapong S. Prevalence of *Fasciola* spp. infections of sheep in the Middle awash River Basin, Ethiopia. Southeast Asian J Trop Med Public Health. 2007; 38 (Suppl.1): 51–57.

[2] Karapınar A Yıldırım A Bişkin Z Düzlü Ö İnci A. The Investigation of Fasciolosis in Sheep by Coproantigen ELISA and Sedimentation-Zinc Sulphate Flotation Technique around Zara Region (Turkey). Kafkas Üniv Vet Fak Derg. 2012; 18: 7–12.

[3] Khoramian H Arbabi M Osqoi MM Delavari M Hooshyar H Asgari M. Prevalence of ruminants fascioliasis and their economic effects in Kashan, center of Iran. Asian Pac J Trop Biomed. 2014; 4 (11): 918–22.

[4] Yavuz A İnci A Yıldırım A İça A Düzlü Ö Distribution of *Fasciola hepatica* in Cattle. Erciyes Üniv Sağlık Bilim Derg. 2007; 16: 96–102.

[5] Batmaz H. Koyun ve Keçilerin İç Hastalıkları. Semptomdan Tanıya, Tanıdan Sağaltıma.: Nobel Tıp Kitabevi;2013.

[6] Celik OY Sayın-İpek DN Aslan-Celik B Irak K Akgül G. Investigation of Seroprevalence of *Toxoplasma gondii* in cattle in Siirt province in Turkey. Indian J Anim Res. 2018; 52 (7): 1053–7.

[7] Irak K Celik BA Karakoc Z Çelik ÖY Mert H Mert N Kaya MO. Oxidant/Antioxidant Status, PON1 and ARES Activities, Trace Element Levels, and Histological Alterations in Sheep with Cystic Echinococcosis. Iran J Parasitol. 2018; 13 (3): 448–56. 30483337 PMC6243163

[8] Caya H. Prevalence of of Helminth Infection of the Liver in Small Animals Slaughtered in the Adana Province. AVKAE Derg. 2012; 2 (2): 12–7.

[9] Soundararajan C Kumar RA Raman M Iyue M. Prevalence of fasciolosis in sheep in Nilgiris. Indıan J Anim Res. 2000; 34 (1): 73–4.

[10] Öge H Tanı Gönenç B. In Tınar R Korkmaz M (Eds.), Fasciolosis İzmir: The Turkish Society for Parasitology. 2003 pp. 135–41.

[11] Meteoroloji Turkish State Meteorological Service. Thornthwaite climate classification. (1938–2017). 2018 [cited 2018 08.25]; https://goo.gl/ud7dBK

[12] Adanır R Çetin H. Prevalence of Liver Flukes in Sheep Slaughtered in Antalya Abbattoir. MAE Vet Fak Derg. 2016; 1 (1): 15–20.

[13] Kaplan M Başpınar S. Incidence of Fasciolosis in Animals Slaughtered in Elâzığ during Last Five Years Period and its Economic Significance. Fırat Med J. 2009; 14 (1): 025–7.

[14] Sahin T. Investigation of some trace element levels and biochemical parameters in sheep with endoparasite. YYU Health Science Institute, PhD. Thesis, Van, Turkey. 2000:

[15] Acici M Buyuktanir O Bolukbas CS Pekmezci GZ Gurler AT Umur S. Serologic detection of antibodies against *Fasciola hepatica* in sheep in the middle Black Sea region of Turkey. J Microbiol Immunol Infect. 2017; 50 (3): 377–81. 26303043 10.1016/j.jmii.2015.06.005

[16] Biçek K Değer S. The Prevalence of Liver Fluke in Sheep and Goats Slaughtered in Tatvan Abbattoir. Van Vet J. 2005; 16 (1): 41–3.

[17] Değer S Akgül Y Ağaoğlu Z Taşçı S. Investigations on the Ecology and Epidemiology of Fascioliasis Infections Resulting from *Fasciola gigantica* in and Around Van. Van Vet J. 1992; 3 (1–2): 133–40.

[18] Gargılı A Tüzer E Gülenber A Toprak M Efil İ Keleş V Ulutaş M. Prevalence of liver fluke infections in slaughtered animals in Trakya (Thrace), Turkey. Turk J Vet Anim Sci. 1999; 23 (2): 115–6.

[19] Gıcık Y Arslan M Kara M Akça A. The prevalence of Liver flukes in Sheep Slaughtered in Kars Province. Kafkas Üniv Vet Fak Derg. 2002; 8 (2): 101–2.

[20] Gül A Aydın A. Prevalence of Liver Flukes in Hair Goats Slaughtered in Hakkâri (Yüksekova) Province. Turkiye Parazitol Derg. 2008; 32 (4): 334–6. 19156606

[21] Kara M Gicik Y Sari B Bulut H Arslan M. A slaughterhouse study on prevalence of some helminths of cattle and sheep in Malatya Province, Turkey. JAVA. 2009; 8 (11): 2200–5.

